# Retest and treat: a review of national HIV retesting guidelines to inform elimination of mother‐to‐child HIV transmission (EMTCT) efforts

**DOI:** 10.1002/jia2.25271

**Published:** 2019-04-08

**Authors:** Alison L Drake, Kerry A Thomson, Caitlin Quinn, Morkor Newman Owiredu, Innocent B Nuwagira, Lastone Chitembo, Shaffiq Essajee, Rachel Baggaley, Cheryl C Johnson

**Affiliations:** ^1^ Department of Global Health University of Washington Seattle WA USA; ^2^ Department of Epidemiology University of Washington Seattle WA USA; ^3^ HIV Department World Health Organization Geneva Switzerland; ^4^ Family and Reproductive Health Cluster World Health Organization, Regional Office for Africa Ouagadougou Burkina Faso; ^5^ HIV/Tuberculosis/Hepatitis Programme World Health Organization, Regional Office for Africa Lusaka Zambia; ^6^ HIV Section UNICEF New York NY USA; ^7^ Clinical Research Department London School of Hygiene and Tropical Medicine London United Kingdom

**Keywords:** HIV, mother‐to‐child transmission, retesting, incidence, prevention of mother‐to‐child HIV transmission

## Abstract

**Introduction:**

High maternal HIV incidence contributes substantially to mother‐to‐child HIV transmission (MTCT) in some settings. Since 2006, HIV retesting during the third trimester and breastfeeding has been recommended by the World Health Organization in higher prevalence (≥5%) settings to reduce MTCT. However, many countries lack clarity on when and how often to retest pregnant and postpartum women to optimize resources and service delivery. We reviewed and characterized national guidelines on maternal retesting based on timing and frequency.

**Methods:**

We identified 52 countries to represent variations in HIV prevalence, geography, and MTCT priority and searched available national MTCT, HIV testing and HIV treatment policies published between 2007 and 2017 for recommendations on retesting during pregnancy, labour/delivery and postpartum. Recommended retesting frequency and timing was extracted. Country HIV prevalence was classified as: very low (<1%), low (1% to 5%), intermediate (>5 to <15%) and high (≥15%). Women with unknown HIV status at delivery/postpartum were included in retesting guidelines.

**Results and discussion:**

Overall, policies from 49 countries were identified; 51% from 2015 or later and most (n = 25) were from Africa. Four countries were high HIV prevalence, seven intermediate, sixteen low and twenty‐two very low. Most (n = 31) had guidance on universal voluntary opt‐out HIV testing at the first antenatal care (ANC) visit. Beyond the first ANC visit, the majority (78%, n = 38) had guidance on retesting; 22 recommended retesting all women with unknown/negative status, five only if unknown HIV status, three in pregnancy based on risk and eight combining these approaches. Retesting was universally recommended during pregnancy, labour/delivery, and postpartum for all high prevalence settings and four of seven intermediate prevalence settings. Five UNAIDS priority countries for EMTCT with low/very low HIV prevalence, but high/intermediate MTCT, had no guidance on retesting.

**Conclusions:**

Retesting guidelines for pregnant and postpartum women were ubiquitous in high prevalence countries and defined in some intermediate prevalence countries, but absent in some low HIV prevalence countries with high MTCT. Countries may require additional guidance on how to optimize maternal HIV testing and whether to prioritize retesting efforts or discontinue universal retesting based on HIV incidence. Research is needed to assess country‐level guideline implementation and impact.

**Abbreviations**
ANC: antenatal care
ART: antiretroviral therapy
EMTCT: elimination of mother‐to‐child HIV transmission
HIV: human immunodeficiency virus
HIVST: HIV self‐test
MCH: maternal and child health
MTCT: mother‐to‐child HIV transmission
PMTCT: prevention of mother‐to‐child HIV transmission
PrEP: pre‐exposure prophylaxis
WHO: World Health Organization

## 1 Introduction

Maternal HIV testing and treatment is the cornerstone of prevention of mother‐to‐child HIV transmission (PMTCT) programmes, and has helped to prevent over two million paediatric HIV infections since 2000 1. Despite implementation of highly effective prevention interventions, in 2017, an estimated 180,000 new paediatric HIV infections occurred; the vast majority (90%) in sub‐Saharan Africa 1. High (>3 per 100 person‐years) maternal HIV incidence (during both pregnancy and breastfeeding) contributes substantially to mother‐to‐child HIV transmission (MTCT) in sub‐Saharan Africa 2, 3, 4, 5, 6, 7, 8, 9, 10, 11, 12, accounting for nearly half of all paediatric HIV infections in some high prevalence settings 13, 14. Thus, efforts to move towards elimination of mother‐to‐child HIV transmission (EMTCT) will increasingly need to focus on prevention, and early identification and treatment of HIV infections acquired during pregnancy and breastfeeding to achieve viral suppression.

Identification of newly HIV‐infected pregnant and postpartum women will be important to achieve the UNAIDS 90:90:90 goals 15. While efforts towards EMTCT encompass both high and low prevalence settings, timely identification and treatment of HIV‐infected pregnant women are critical components of all successful programmes. As a result, most countries have implemented maternal HIV testing as a routine component of antenatal care (ANC) services to maximize prevention potential through early maternal treatment and infant prophylaxis 16. However, strategies to detect HIV infections among women who miss ANC testing or initially test HIV negative during pregnancy and acquire HIV either later in pregnancy or while breastfeeding are also needed. Women who have difficulty accessing healthcare services, or were not offered HIV testing during ANC due to lack of counselling or HIV test kit stock‐outs, have increased risks of not knowing their HIV status 17, 18. Retesting during pregnancy/postpartum has been shown to be an effective strategy to detect incident maternal infections 3, 4, 9, resulting in low MTCT transmission rates in one study conducted in Kenya 3.

Since 2006, the World Health Organization (WHO) has recommended retesting pregnant women for HIV in countries with high prevalence during the third trimester, and since 2015 has expanded that guidance to include retesting during pregnancy, at labour/delivery and/or in the postpartum period 19, 20, 21. In low prevalence countries and countries with concentrated epidemics, maternal retesting is recommended for women at high risk in serodiscordant relationships or members of key populations 19, 20, 21, 22, 23. The lack of clarity on the specific frequency and time points to conduct retesting during pregnancy and the postpartum period can create confusion for programme implementers about when to offer retesting, contributing to suboptimal implementation. In contrast, other programmes may be offering retesting more frequently than needed, and wasting limited public health resources on unnecessary retesting 22, 24, 25. Countries need additional data on the impact of retesting during pregnancy, labour/delivery and postpartum to appropriately allocate HIV resources, including allocation within PMTCT programmes. Many countries have national guidelines on maternal HIV retesting, but similarities and differences between guidelines based on geographic region, HIV prevalence, MTCT rates or country‐level priorities for EMTCT have not been previously described.

We reviewed national HIV guidelines on HIV testing for pregnant and postpartum women to characterize current recommendations for the timing and frequency of maternal HIV retesting. In addition, we compared retesting strategies by geographic region, national HIV prevalence and MTCT rates.

## 2 Methods

### 2.1 Guideline selection, review process and translations

We conducted a standardized review of individual country policies on maternal HIV retesting during pregnancy and postpartum. A total of 52 countries were selected to review, representing a range of HIV prevalence, geographic regions and all 23 UNAIDS priority countries for EMTCT 26, which collectively account for the majority of HIV infections among pregnant women. UNAIDS has prioritized countries for EMTCT due to the high number of HIV infections among women, children and adolescents 26. Countries with available guidelines on PMTCT, antiretroviral treatment (ART), and/or HIV testing from 2007 (following initial WHO recommendations) or later were included in the review. Any country guidelines on HIV self‐testing or pre‐exposure prophylaxis (PrEP) were also reviewed when available. Guidelines were requested directly from country contacts via email if they were not previously identified for inclusion of a prior WHO HIV testing policy review or available through online searches 27. We used the summary of the guidelines provided by country contacts if they were able to provide documentation of their guidelines along with the English summary. In addition, country contacts who confirmed that no retesting guidelines exist were included in our review and countries were classified as not having retesting guidelines. Guidelines published in 2012 or later were prioritized for review, and countries were contacted to request updated guidelines if publication dates were between 2007 and 2011. For countries with multiple available guidelines, we used the most recent published guideline, or the guideline with the most complete information on maternal HIV testing policies.

Guidelines were reviewed for sections relevant to HIV testing during pregnancy and postpartum, and using a key word search for the following terms: pregnancy, antenatal, labor/labour, delivery, postpartum, postnatal, breastfeeding and/or lactating. A primary reviewer extracted data from the guidelines into a standardized extraction tool; a secondary reviewer repeated this process and highlighted any discrepancies with the primary reviewer (Data S1). Discrepancies were discussed and resolved between primary and secondary reviewers, or with a third reviewer, if needed.

No country guidelines were excluded from the current review based on language. Non‐English languages included Chinese, Portuguese, French, Spanish and Ukrainian. All non‐English guidelines (except from Ukraine and Cuba) were reviewed by an independent translator using only key word searches; text related to HIV testing and the key word(s) identified was extracted into a standardized form (referencing the page number with original text) and translated into English. Translations of guidelines from Ukraine and Cuba were summarized by a country contact. A secondary reviewer fluent in the original language also reviewed the guidelines and translated text to ensure all references to maternal HIV testing were extracted and accurately translated.

Data extracted from country guidelines included initial ANC HIV testing; guidelines for HIV retesting during pregnancy, labour/delivery and postpartum/breastfeeding; timing and eligibility for HIV retesting; and frequency of retesting. Since women who missed initial HIV testing during pregnancy for any reason (e.g. test kit stock‐out, declined test and/or did not access ANC) would programmatically be captured in retesting programmes, we extracted recommendations on HIV testing for women who received or missed initial HIV testing during pregnancy and classified this as maternal retesting.

Guidelines were classified as having any guidance on maternal HIV retesting (yes/no). Countries with general population policies for retesting that were not specific to pregnant or postpartum women were classified as not having guidance on maternal HIV retesting. Countries with maternal HIV retesting guidelines were classified based on their approach to retesting as: (1) *universal* if testing recommendations apply to all pregnant and/or postpartum women; (2) *targeted* if testing recommendations apply to pregnant and/or postpartum women based on individual‐ or population‐level risk factors; (3) *only if unknown* if testing recommendations are only for pregnant/postpartum women without documented HIV test results from earlier ANC visits; (4) *combination* if more than one approach was recommended during pregnancy and postpartum (universal, targeted and/or only if unknown approaches; Table 1). Individual or population‐level risk factors for targeted retesting recommendations included belonging to a group with high risk of HIV exposure, being in an HIV serodiscordant relationship, or residing in a high HIV prevalence setting. Universal, combination, or only if unknown approaches to HIV testing were all considered population‐level approaches to retesting (vs. targeted only approaches only). Approaches to maternal HIV retesting were stratified based on the time period (general peripartum, pregnancy, labour/delivery and/or postpartum/breastfeeding) and frequency (1 or >1 retest per time period) of maternal retesting.

**Table 1:** Definitions for Country approaches to maternal HIV retesting

Definition of approach	Populations recommended to receive maternal HIV retesting
Universal	All pregnant/postpartum women
Targeted^a^	Dependent on risk behaviour, partner status, and/or high HIV prevalence
Only if unknown	Only if maternal HIV status is unknown/no previous HIV test result documented
Combination	Combination of universal, targeted, and/or only if unknown approaches, depending on peripartum status

a Individual‐level approach.

Countries were classified according to WHO regions (Americas, Africa, South‐East Asia, Europe, Eastern Mediterranean or Western Pacific). Country‐level HIV prevalence in the general population data was abstracted from 2016 UNAIDS estimates if available, or other published estimates from country guidelines. Estimated rates of MTCT (including the breastfeeding period up to 36 months) per country were obtained from the 2016 UNAIDS Spectrum model estimates or other published sources. We developed categories for both HIV prevalence and MTCT as follows: very low (<1%), low (1% to 5%), intermediate (>5% to <15%) and high (≥15%) based on previous work by Ishikawa and colleagues 28.

## 3 Results and discussion

### 3.1 Country characteristics

Among 52 countries selected for the review, 49 countries with available guideline documents were successfully retrieved (including two countries who confirmed the absence of maternal retesting guidelines) and included in the review. Three countries with unavailable guidelines and no communication from countries about retesting included: Belarus, Burkina Faso and Comoros. Most (n = 25) countries were from the African Region, followed by the Eastern‐Mediterranean (n = 7), Americas (n = 6), South‐East Asia (n = 5), Western‐Pacific (n = 4) and European (n = 2) regions. The review included four high prevalence (≥15%), seven intermediate prevalence (>5 ‐ <15%), sixteen low prevalence (1% to 5%) and twenty‐two very low prevalence (<1%) countries. Twenty‐three (47%) countries are classified as UNAIDS priority countries for EMTCT. English language guidelines were available for full text review for 34 countries; 11 countries with non‐English guidelines had limited key word text review after translation. In addition, two country contacts provided a summary of guidelines translated into English (Ukraine and Cuba) and two country contacts confirmed a lack of retesting maternal guidelines (Morocco and Vietnam).

### 3.2 Overview of current maternal retesting guidelines

**Figure 1:**
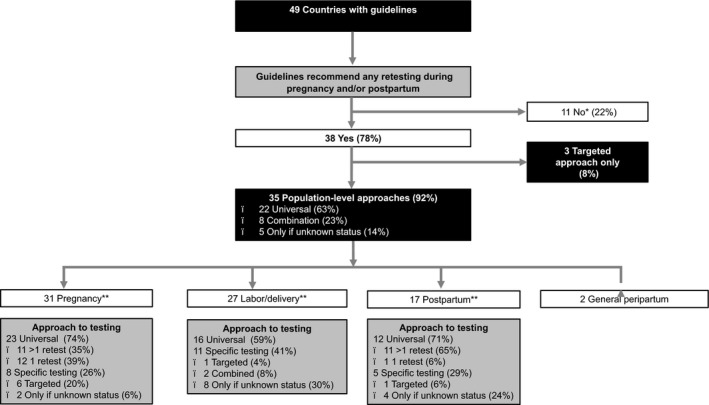
Maternal HIV retesting recommendations in 49 HIV policies *Includes 1 country (Indonesia) where guidance follows general population *approach only*. **Not mutually exclusive. Universal; retesting recommended for all pregnant and/or postpartum women. Targeted; retesting recommended based on individual or population‐level risk factors. Only if unknown; retesting recommended only if maternal HIV status is not previously documented. Combination; retesting recommended using a combination of universal, targeted and unknown approaches depending on the peripartum stage. General peripartum includes Ethiopia and Tanzania.

Most countries had guidance specifying universal voluntary opt‐out HIV testing at the first ANC visit (n = 31). Beyond the initial HIV test, the majority of countries (n = 38) had guidance on maternal HIV retesting (Figure 1). Eleven countries, including five priority EMTCT countries, were classified as not having maternal retesting guidelines: four countries with low HIV prevalence (Burundi, Chad, Cote d'Ivoire, and Trinidad and Tobago), and seven countries with very low HIV prevalence (Australia, Democratic Republic of the Congo, Indonesia, Morocco, Philippines, Somalia and Vietnam).

Three countries use a targeted approach for maternal retesting. El Salvador recommends retesting during each trimester of pregnancy and at labour/delivery if women have a high risk of HIV acquisition, including members of HIV serodiscordant couples; Pakistan recommends retesting for pregnant women with known or continued HIV exposure; and Sierra Leone recommends retesting for pregnant women 6 to 12 weeks after the initial test in case they were tested during the window period of detection or if they had an additional exposure to HIV.

The majority (n = 35) of countries recommend a population‐level testing approach, most commonly a universal approach (n = 22, 58%). Eight countries (21%) followed a combination approach, employing both universal and targeted approaches. In five very low HIV prevalence countries (Afghanistan, Cambodia, Egypt, Nepal and Sudan), maternal retesting is not targeted based on individual‐ or population‐based HIV risk but only offered if the woman's status is unknown, that is, initial ANC HIV testing was not conducted.

In two low HIV prevalence countries, Ethiopia and Tanzania, maternal retesting is advised but guidelines lack specificity on the frequency and timing of retesting. Table 2 presents an overview of the testing approach, frequency and recommended time points for maternal retesting by country among the 38 countries that recommend any maternal HIV retesting. Among countries recommending retesting during pregnancy and/or postpartum, the most commonly targeted time points are during the third trimester of pregnancy, at six weeks postpartum and/or every three months during breastfeeding.

**Table 2:** Testing approach, frequency and targeted time points among countries with guidance on maternal HIV retesting (N = 38)

Country information				Retesting approaches and peripartum status			
WHO Region	Country^a^^,^ b	HIV Prevalence^c^	MTCT^d^	Approach^e^	Pregnancy	Labour/delivery	Postpartum
African (n = 21)	Angola 29, a ^,^ b	1.9%	21.0%	Universal	Each trimester	Yes	General population
	Botswana 30, a	21.9%	4.8%	Universal	Every three months	Yes	Six weeks, every three months if BF
	Cameroon 31, a	3.8%	12.8%	Universal	16 to 28, 28 to 36, 36 to 42 weeks	Yes	Day 6, then monthly six weeks to six months (only if unknown or last negative test >3 months prior)
	Central African Republic 32, b	4.0%	11.7%	Combination	Third trimester	Only if previous negative test >3 months	Every three months
	Ethiopia 33	1.1%	15.9%	General peripartum	‐	‐	‐
	Ghana 34, a	1.6%	17.7%	Combination	Third trimester	Only if unknown	Only if unknown
	Kenya 35, a	5.4%	8.3%	Universal	Third trimester	Yes	Six weeks, six months
	Lesotho 36, a	25.0%	11.0%	Universal	36 weeks	Yes	Every three months during BF
	Liberia 37	1.6%	16.3%	Universal	Third trimester	Yes, general inpatient guidelines	Yes, during <5 years MCH services
	Malawi 38, a	9.2%	7.8%	Universal	Every three months	Yes	Every three months
	Mozambique 39	12.3%	11.1%	Universal	Every three months	No	No
	Namibia 40, a	13.8%	4.2%	Universal	Every three months; thirty‐six weeks	No	Six weeks and every six months during BF
	Nigeria 41, a	2.9%	21.6%	Universal	Third trimester	Yes	No
	Rwanda 42	3.1%	7.6%	Combination	Every three months if serodiscordant relationship	Yes	Every three months though twenty‐four months if serodiscordant relationship
	Sierra Leone 43	1.7%	9.0%	Targeted^f^	Six to twelve weeks after initial test	No	No
	South Africa 44, a	18.9%	4.6%	Universal	Each ANC visit	Yes	Every three months during BF
	Eswatini (Swaziland) 45, a	27.2%	6.0%	Universal	Eight weeks after initial test, then every ANC (emphasis on Third trimester)	Yes	Six weeks, then every visit during BF (emphasis on child vaccination schedule)
	Tanzania 46	4.7%	11	General peripartum	‐	‐	‐
	Uganda 47, a	6.5%	4.1%	Universal	Third trimester	Yes	Every three months during BF
	Zambia 48, a	12.4%	10.7%	Universal	Every three months	Yes	Six weeks and every three months during BF
	Zimbabwe 49, a	13.5%	7.0%	Universal	Third trimester	Yes	Six weeks and every six months during BF, especially at nine months
Americas (n = 5)	Brazil 50, b	0.6%	6.0%	Only if unknown	No	Only if unknown	No
	Canada 51	0.2%	NA	Combination	Only if high risk	Only if unknown	No
	Cuba 52, 53, g	0.4%	1%	Universal	Second and third trimester^g^	Only if unknown	Only if unknown
	El Salvador 54, b	0.6%	15.0%	Targeted (high risk)	Second and third trimester	Yes	No
	Haiti 55	2.1%	10.1%	Combination	Third trimester if high risk of HIV status unknown	Only if unknown	Only if unknown
Eastern Mediterranean (n = 5)	Afghanistan 56	0.09%	30.1%	Only if unknown	Only if unknown	Only if unknown	No
	Egypt 57	0.09%	24.7%	Only if unknown	No	Only if unknown	No
	Pakistan 58	0.1%	31.2%	Targeted	If prior or continued exposure	No	No
	Sudan 59	0.2%	29.4%	Only if unknown	Only if unknown	Only if unknown	Only if unknown
	Tunisia 60	0.09%	NA	Universal	Six months gestation	No	No
Europe (n = 2)	Ukraine 61, 62, 63, b	0.9%	13.9%	Combination	If high risk/serodiscordant, timing NS	Only if unknown or high risk	No
	United Kingdom 64	0.019%	NA	Combination	If high risk, timing NS	Only if unknown or high risk	No
South East Asia (n = 4)	Cambodia 65	0.6%	10.3%	Only if unknown	No	Only if unknown	No
	India 66, a	0.3%	NA	Combination	If high risk, timing NS	Only if unknown	No
	Nepal 67	0.2%	17.6%	Only if unknown	No	No	Only if unknown
	Thailand 68	1.1%	1.0%	Combination	Universal 28 to 32 weeks, if high risk use 4th generation test and repeat in two weeks	Yes	Every six months, if high risk and on PrEP follow PrEP protocol
Western Pacific (n = 1)	China^b^ ^3^	0.004%	NA	Universal	No	Yes	No

ANC, antenatal care; BF, breastfeeding; MCH, maternal and child health; MTCT, mother‐to‐child HIV transmission; NA, not applicable; NS, not specified; PrEP, pre‐exposure prophylaxis.

^a^UNAIDS priority country for elimination of mother‐to‐child transmission (EMTCT); ^b^limited review of non‐English guidelines; ^c^HIV prevalence estimates in general population from UNAIDS 2016; ^d^estimated mother‐to‐child HIV transmission from UNAIDS SPECTRUM model; ^e^ *Universal:* retesting recommended for all pregnant and/or postpartum women; *targeted*: retesting recommended based on individual or population‐level risk factors; *only if unknown:* retesting recommended only if maternal HIV status is not previously documented; *combination*: retesting recommended using a combination of universal, targeted, and unknown approaches depending on the peripartum stage; ^f^if in window period or additional exposure; ^g^M. de Mello (personal communication, Aug 7, 2018), retesting guidance disseminated in 2017 was used to categorize retesting in pregnancy. Guidelines from 2012 indicate retesting during pregnancy only in the third trimester.

### 3.3 Recommended timing and frequency of HIV retesting

**Figure 2:**
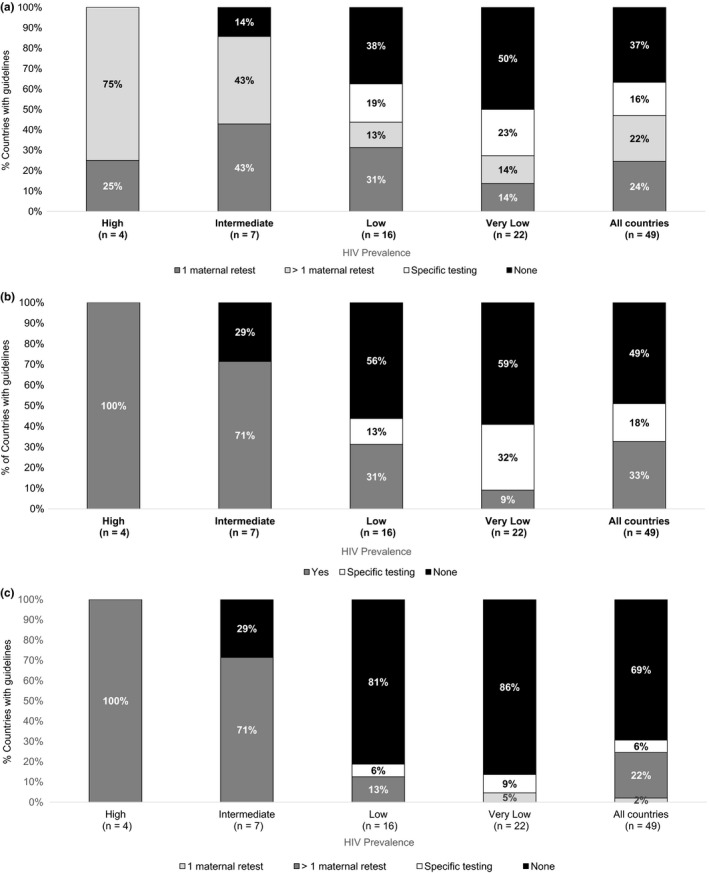
Maternal HIV retesting approaches among 49 countries, by HIV prevalence HIV prevalence in general population defined as high (≥15%), intermediate (>5% to <15%), low (1% to 5%%) or very low (<1%). Universal testing for all pregnant women or all postpartum women is categorized as 1 or >1 retest. Specific testing includes: targeted guidelines based on risk, only if unknown, or combination approaches. **(a)** During pregnancy. **(b)** During labour/delivery. **(c)** During the postpartum period.

Among all 49 countries reviewed, the majority (n = 31, 63%) recommend retesting during pregnancy. Twenty‐three countries recommend a universal testing approach, including 1 retest (n = 12, 24%) or >1 retest (n = 11, 22%); and eight countries (16%) recommend maternal retesting based on targeted risk factors, unknown status or a combination of factors (Figure 2a). Most countries (n = 27, 55%) recommend retesting during labour/delivery. Sixteen countries (33%) recommend a universal HIV testing strategy for all women during labour/delivery and 11 (22%) recommend maternal retesting based on targeted risk factors, unknown status or a combination of factors (Figure 2b). Similarly, one third of countries (n = 17, 35%) recommend maternal retesting in the postpartum period. Eleven countries (22%) recommend a universal HIV testing strategy for all postpartum women at more than one time point, 1 (2%) recommend retesting once during the postpartum period, and four countries (10%) recommend maternal retesting based on targeted risk factors or unknown status (Figure 2c).

Among the 33 countries that had recommendations on the timing of retesting during the paripartum period, 15 (45%) recommend retesting during pregnancy, labour and delivery, and postpartum; 5 (15%) in pregnancy only; 4 (12%) at labour and delivery only; 1 (3%) only postpartum; 7 (21%) in pregnancy and at labour and delivery; and 1 (3%) in pregnancy and postpartum. Eswatini (formerly Swaziland) and South Africa, where HIV prevalence is 27% and 18.9% respectively, have guidelines recommending some of the most frequent maternal retesting. Eswatini recommends retesting eight weeks after the initial test, at each subsequent ANC visit, during labour/delivery, at six weeks postpartum, and every clinic visit during breastfeeding. Similarly, in South Africa retesting is recommended at each ANC visit, during labour/delivery and every three months until cessation of breastfeeding. As South Africa is moving to ANC visits monthly until the third trimester, and bi‐weekly during the third trimester, this could constitute eight to ten retests during pregnancy for women depending if ANC is initiated early, together with our retests during delivery/postpartum, for a total of 14 tests.

**Figure 3:**
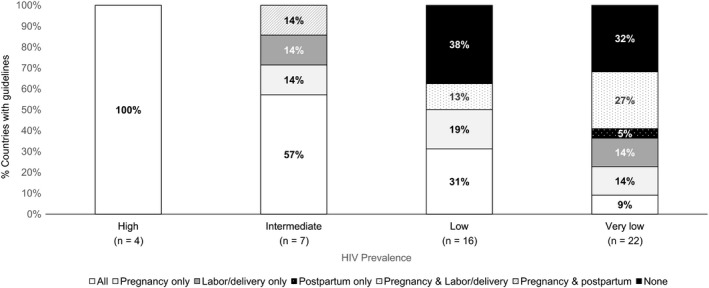
Frequency of maternal HIV retesting among 49 countries, by HIV prevalence All includes recommendations during pregnancy, labor/delivery, and the postpartum period. HIV prevalence in general population defined as high (≥15%), intermediate (>5% to <15%), low (1% to 5%) or very low (<1%).

There was a clear relationship between HIV prevalence and retesting guidance, with recommendations to conduct retesting more frequently as country HIV prevalence increased at each time point (pregnancy, labour/delivery and postpartum) (Figure 3). Retesting guidelines were ubiquitous in all four high HIV prevalence countries and four of seven intermediate prevalence countries, with a universal approach to retesting recommended at multiple time points during pregnancy, labour/delivery and in the postpartum period. The majority (63%, n = 10) of low HIV prevalence settings did have retesting recommendations; 31% (n = 5) recommended retesting during pregnancy, labour/delivery and postpartum; 19% only during pregnancy, 13% during pregnancy and labour/delivery. While most (68%, n = 15) countries in very low HIV prevalence settings had recommendations for retesting, there was substantial heterogeneity in the approach to testing between countries. Only 14% (n = 3) of very low HIV prevalence settings had any guidance on retesting in the postpartum period.

**Figure 4:**
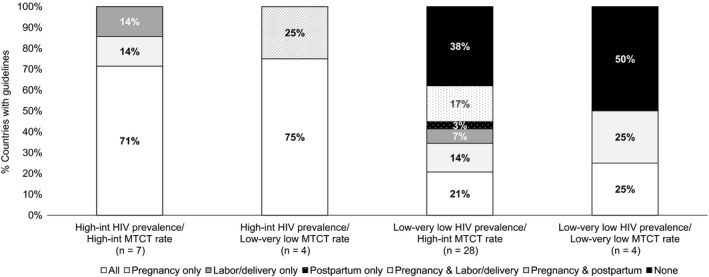
Maternal HIV retesting among 44 countries, by HIV prevalence and mother‐to‐child HIV transmission rate All includes recommendations during pregnancy, labour/delivery and the postpartum period. MTCT; mother‐to‐child HIV transmission. MTCT rate and HIV prevalence classified as High‐int (intermediate) if >5%; Low‐very low if ≤5%. Five countries missing MTCT rate excluded from this analysis.

Data on MTCT rates from 2016 were available for 44 countries included in the policy review. Among 33 of these countries with low/very low HIV prevalence, 29 had high/intermediate MTCT rates of which 38% (n = 11) did not have any recommendations for maternal HIV retesting during pregnancy, labour and delivery, or in the postpartum period (Figure 4) despite seven being EMTCT priority countries. Low/very low HIV prevalence countries with high/intermediate MTCT rates who lack maternal retesting guidance may consider recommending a maternal HIV retest to reduce MTCT rates attributable to incident maternal infections, particularly for women who may have an elevated risk of HIV acquisition. In contrast, among 11 high/intermediate HIV prevalence countries, four countries (all were EMTCT priority countries) had low/very low MTCT rates and 100% recommended retesting at least twice during pregnancy and postpartum (at least two total retests). Despite the high/intermediate HIV prevalence of these countries, increasing the number of times women are retested may have limited utility in these settings and may indicate that pregnant and postpartum women are being over‐tested. Reducing or eliminating maternal retesting in these settings may be appropriate, allowing maternal retesting resources to be reallocated towards other HIV treatment and prevention strategies. Mathematical models to assess the cost‐effectiveness of maternal retesting in countries with low HIV prevalence or low MTCT rates are needed to help determine the public health and economic impact of modifying retesting policies in specific settings.

Our review was subject to some limitations. We were unable to obtain guidelines for three countries and may not have the most recent versions of retesting guidelines for some countries. National programmes may be in the process of guideline revision, or implementing practices that have not yet been incorporated into written national policies, and our results would not reflect these changes. Furthermore, we did not summarize national policies in countries with concentrated epidemics in key populations due to the low frequency of these types of policies.

Written guidelines supporting maternal retesting for HIV are important to detect new maternal HIV infection and move towards EMTCT. However, it is also important to measure guideline implementation and assess how guidelines translate into public health practice. Several barriers to HIV testing have previously been described, including difficulty accessing HIV counselling and testing services, stigma, test kit stock‐outs, inaccurate perceptions of HIV risk and fear of positive test results 69. While these barriers also apply to HIV retesting, there are additional barriers unique to retesting. Healthcare workers may have difficulty tracking guidelines for different populations, or may not be aware that guidance is dependent on individual‐ or population‐level characteristics. In addition, vague language in guidelines about who and when to retest can result in missed opportunities to offer peripartum women retesting. Prior studies in sub‐Saharan Africa suggest that only ~30% of pregnant women eligible for retesting who returned to the clinic received a test 70, 71. In contrast, implementing retesting guidelines designed for the general population can also create confusion for healthcare workers to retest pregnant and postpartum women. While attendance at maternal and child health (MCH) clinics for ANC, postnatal care and infant immunizations is high in many settings, the MCH visit schedule does not follow regular intervals making it more challenging to align calendar interval recommendations for retesting (e.g. every six months) with MCH visits. Furthermore, in many settings the only time during the postpartum period where maternal health is specifically assessed is at four to six weeks postpartum; all other visits are related to infant health.

Use of HIV self‐tests (HIVSTs) may be one approach to overcome challenges with clinic‐based retesting for pregnant and postpartum women, as well as testing partners. Reaching partners of pregnant and postpartum women through partner HIV testing and partner notification services remain crucial to HIV testing approaches and efforts to reduce new maternal infections. Some studies have found high acceptability of oral HIVST among male partners given test kits by pregnant or postpartum partners through a “secondary distribution” strategy from MCH clinics 72, 73. This approach relies on instructing women how to use HIVST, including how to interpret results and referral procedures for confirmatory testing if initial HIVST results are positive. However, recent analyses continue to show that both policies to support partner HIV testing and partner notification, and implementation of these activities, are lacking 74. In contrast, evaluations of HIVST to support maternal HIV retesting have not been conducted. It is possible that home‐based HIVSTs, using either saliva or blood, could be offered as an additional, available approach to overcome some of the barriers to clinic‐based blood HIV testing and increase retesting rates among women who may otherwise fail to be tested again. Alternatively, clinic‐based HIVST could provide an opportunity to reduce healthcare system burden to conduct maternal retesting for triage, while also offering women privacy and confidentiality outside of their homes. In our review, we systematically searched for guidance on HIVST, and while HIVST is available and recommended for general population use in some countries, no country has yet endorsed HIVST as a strategy to increase HIV retesting for pregnant and postpartum women.

While improving HIV testing services is a critical component of HIV prevention for pregnant and postpartum women, complementary approaches including screening for sexually transmitted infections (STIs), condom promotion and PrEP will also be important to prevent new HIV infections in mothers and infants. Recent WHO recommendations supporting PrEP use during pregnancy and breastfeeding 75 following evidence of PrEP safety 76, 77 have led to pilot studies of PrEP implementation in ANC and PNC settings 78, but the impact PrEP will have on reducing new maternal infections and MTCT is unknown. Programmes should continue to support comprehensive HIV prevention strategies during pregnancy and postpartum, including maternal retesting for HIV, based on epidemic context.

## 4 Conclusions

Maternal HIV retesting will increasingly be important to reach EMTCT targets by detecting new maternal HIV infections and achieving the 90:90:90 targets 15, including maternal viral suppression through the linkage of newly diagnosed HIV‐infected women to HIV care and treatment. Globally, young women of reproductive age have the highest risks of HIV acquisition and strategies like maternal HIV retesting will also be critical to interrupt heterosexual transmission as other prevention interventions are rolled out. Countries with high HIV prevalence universally recommended retesting pregnant and postpartum women, but some low HIV prevalence countries with high MTCT rates lack any guidance on maternal retesting. Countries may find it helpful to receive guidance on whether retesting should be offered to pregnant and/or postpartum women, and the timing of retesting for countries that view retesting as essential to capturing maternal incidence. Furthermore, while wider implementation of PrEP during pregnancy and postpartum over time may alter approaches and necessity for maternal HIV retesting, it will be important to understand context specific risks for maternal HIV acquisition and impact of retesting strategies before and after PrEP implementation to guide policies and programmes. Studies measuring the implementation of maternal retesting HIV guidelines, and impact of different testing approaches in various epidemic settings are needed to maximize efforts to detect and treat new maternal HIV infection, while balancing costs of these approaches.

## Competing interests

The authors declare that they have no competing interests.

## Authors’ contributions

ALD, KAT and CQ reviewed and extracted guidelines. ALD, KAT and CJ designed the protocol. SE, RB and CQ reviewed and commented on the protocol. ALD and KAT analysed the data. ALD and KAT wrote the manuscript. CQ, SE, MON, CL, IN, RB and CJ all provided substantial input and edits to the manuscript. All authors have read and approved the final manuscript.

## Supporting information


**Data S1.** Maternal HIV Retesting Extractor_09Oct2018.
Data extraction tool to collect and synthesize data on maternal HIV retesting guidelines, mother‐to‐child HIV transmission, and HIV prevalence.
